# Socioeconomic status, disease duration, and DLQI scores in adults with moderate-to-severe atopic dermatitis: A multicenter cross-sectional study

**DOI:** 10.1016/j.jdin.2026.05.012

**Published:** 2026-05-20

**Authors:** Shrichand G. Parasramani, Rickson Pereira, Anil Ganjoo, Divya Gupta, Maitreyee Panda, Maya Vedamurthy, Supratim Karmakar, Vijay Bhasker Reddy, Vinay Singh, Devesh Joshi, Anoop K, Sheikh Shahid, Noushar Bin Farook V V, Reema Mathai Alapputhara, Dhiraj Dhoot, Tripti Sharma, Ashwin Balasubramanian, Saiprasad Patil, Hanmant Barkate

**Affiliations:** a Consultant Dermatologist, Department of Dermatology, Leelavati Hospital, Mumbai, India; b Consultant Dermatologists, Dr Rickson’s Dermatherapie Clinic, Mumbai, India; c Sr. Consultant Dermatologist & Laser Surgeon, Director, Skinnovation Clinics, New Delhi, India; d Associate Professor, Department of Dermatology, Dr. B.R. Ambedkar Medical College, Bangalore, India; e Professor of Dermatology, Department of Dermatology, Institute of Medical Science and SUM Hospital, Bhubaneswar, Odisha, India; f Director and Chief Dermatologist in RSV Skin Clinic, Chennai, India; g Consultant Dermatologist, Wizderm Clinic, Kolkata, India; h Department of Dermatology, Professor & HOD at Kamineni Institute of Medical Sciences, Director and Chief Dermatologist at CSR Clinics, Telangana, India; i Senior Consultant in Dermatology & Aesthetic Medicine, Director at Vibrance Wellness Vista; j Department of Global Medical Affairs, Glenmark Pharmaceuticals Limited, Mumbai, India

**Keywords:** atopic dermatitis, Dermatology Life Quality Index (DLQI), epidemiology, India, occupation, quality of life, real-world evidence, socioeconomic status

*To the Editor:* Atopic dermatitis (AD) is a chronic inflammatory skin disorder with a well-recognized impact on physical comfort, psychosocial wellbeing, and daily functioning.^1^ While disease severity scoring systems effectively quantify clinical activity, they frequently fail to capture the lived burden experienced by patients. Patient-reported outcome measures such as the Dermatology Life Quality Index (DLQI), therefore play a critical role in comprehensive AD assessment.^2^

With this objective, we conducted a multicentre, cross-sectional Indian study evaluating quality of life (QoL) and its socio-demographic determinants among adults with moderate-to-severe AD in 660 patients across 20 dermatological centres after obtaining ethics approval.

Missing data reflected incomplete routine documentation across centres. An available-case approach was used, and missingness may introduce bias in interpretation. The median DLQI score was 10 (interquartile range 9), indicating a moderate impact on QoL. Notably, nearly two-thirds of patients reported moderate to very large impairment, and 8.6% experienced an extremely large impact. These findings reaffirm that AD exerts a substantial day-to-day burden even among patients receiving routine dermatological care.

DLQI varied by socioeconomic status (SES) and occupations in unadjusted analyses; patients from low SES backgrounds had significantly higher DLQI scores compared with those from medium and high SES groups (Table I). Occupational role was also associated with DLQI; individuals in active work or study roles reported greater impairment than retired individuals. Additionally, longer disease duration was associated with higher DLQI scores, suggesting cumulative psychosocial and functional burden over time (Fig 1). These observations align with global evidence demonstrating substantial quality-of-life burden in adults with AD.^3^^,^^4^

**Table I:** DLQI by socioeconomic status and occupation

Group	*n*	Median (IQR)	*P*-value
Socioeconomic status (*N* = 621)			
Low	162	11.5 (7.0-17.0)	<.001
Medium	304	9.0 (5.0-14.0)	
High	155	8.0 (5.0-12.5)	
Occupation (*N* = 660)			
Student	259	10 (5-14)	<.01
Employed/Professional	222	10 (6-14)	
Housewife/Homemaker	103	10 (6-16)	
Manual labor	39	10 (6-13)	
Retired individuals	37	5 (3-9)	

Nonparametric tests used due to non-normal distributions; Post-hoc (Bonferroni): low vs medium (*P* = .007) and low vs high (*P* < .0001) were significant; medium vs high NS (*P* = .118); For occupation, retired individuals had lower DLQI than employed/professional (*P* = .0014), housewife/homemaker (*P* = .0012), manual labor (*P* = .028), and student (*P* = .0030); other pairwise contrasts NS after correction.

**Fig 1:**
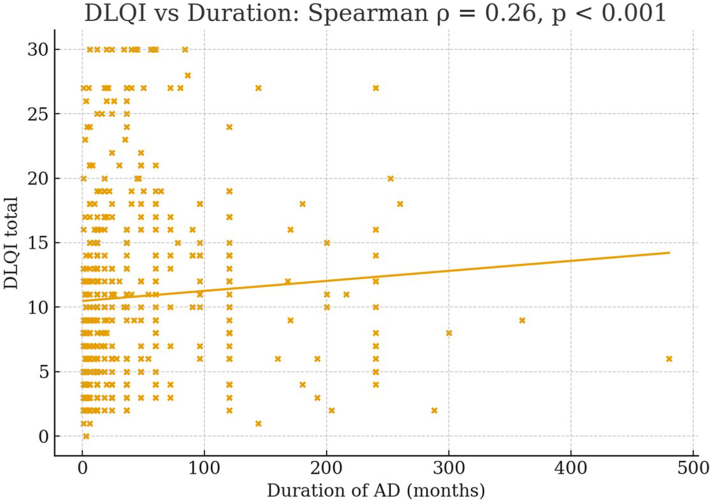
DLQI by duration of disease in months (*n* = 508).

The study underscores the importance of routine DLQI screening in everyday practice and supports its complementary use alongside physician-rated severity indices such as Eczema Area and Severity Index (EASI) or Scoring of Atopic Dermatitis (SCORAD). Incorporating DLQI into routine consultations can help clinicians identify patients who may require additional counselling, psychosocial support, workplace or educational accommodations, and individualized treatment planning.

While the cross-sectional, retrospective design and reliance on routinely recorded data limit causal inference, the multicentre real-world nature of the study enhances its external validity and clinical relevance. Findings should be interpreted cautiously, as unmeasured factors such as disease severity, treatment access, site-level variation, and the absence of clinician-rated severity indices (eg, EASI, SCORAD) may have contributed to the observed differences. Accordingly, the results should be regarded as descriptive, hypothesis-generating, and over-interpretation of unadjusted comparisons are cautioned against. Costs and affordability, work or school impact, occupational stress, and access barriers were not assessed and therefore cannot be inferred. Future studies, especially in low-and-middle-income countries, should capture these domains alongside objective severity measures to evaluate independent associations with DLQI.

In conclusion, the study provides timely evidence that atopic dermatitis significantly impairs QoL in Indian adults, with higher DLQI scores observed in lower-SES participants and lower scores among retired participants, although these associations may be confounded due to unadjusted analyses. Recognizing and addressing these social dimensions through routine DLQI assessment may help move AD management toward a more patient-centered and equitable care model.

## Conflicts of interest

Dr Devesh Joshi, Dr Anoop, Dr Sheikh Shahid, Dr Noushar Bin Farook, Dr Reema Mathai Alapputhara, Dr Dhiraj Dhoot, Dr Tripti Sharma, Dr Ashwin Balasubramanian, Dr Saiprasad Patil, and Dr Hanmant Barkate are employees of Glenmark Pharmaceuticals Limited. Parasramani, Pereira, Ganjoo, Gupta, Panda, Vedamurthy, Karmakar, Reddy, and Singh have no conflicts of interest to declare.
